# Tracing Toxicity: A Scientometric Perspective on Three Decades of Metallic Pollutants Research

**DOI:** 10.1155/jt/8884503

**Published:** 2026-04-08

**Authors:** Prasanna Devi Balachandran, Rajeswari Shanmugam, Vijayalakshmi Varadarajan, Brindha Rethinam, Sankar Ganesh Palani

**Affiliations:** ^1^ Department of Biotechnology, University College of Engineering, BIT Campus, Anna University, Tiruchirappalli, Tamilnadu, India, annauniv.edu; ^2^ Department of Library, University College of Engineering, BIT Campus, Anna University, Tiruchirappalli, Tamilnadu, India, annauniv.edu; ^3^ Department of Biotechnology, Vel Tech High Tech Dr. Rangarajan Dr. Sakunthala Engineering College, Avadi, Chennai, Tamilnadu, India, velhightech.com; ^4^ Department of Biological Sciences, Birla Institute of Technology and Science, Hyderabad Campus, Pilani, Telangana, India, bits-pilani.ac.in

**Keywords:** environmental pollution, heavy metals, Metallic pollutants, scientometric analysis, toxicity, VOSviewer

## Abstract

Rapid industrialization and urbanization have led to the unchecked discharge of toxic metals, posing serious threats to ecosystems and public health. This study analyses scientometric data on toxic metal pollutants, covering publications from 1992 to 2024. About 220 documents (183 research articles and 37 reviews) were extracted from Web of Science using BibExcel and VOSviewer to explore global research trends on metal toxicity, focusing on lead (Pb), mercury (Hg), cadmium (Cd), arsenic (As), chromium (Cr), scandium (Sc), beryllium (Be), and aluminum (Al). A notable rise in research since 2000 reflects growing awareness of metal‐related risks. China leads in publication output, while the United Kingdom ranks highest in citation impact. The National Natural Science Foundation of China (NSFC) played a key role in advancing high‐impact studies. It prioritizes funding for research in environmental toxicology and health effects of pollutants, environmental exposure and health effects of emerging toxic substances, toxicology of micro‐/nanomaterials, toxicological mechanisms and health impacts of atmospheric fine particulate matter (PM_2_._5_), and prevention and control of hazardous chemicals. Keyword co‐occurrence and cluster analysis highlight themes such as bioremediation, nanomaterials, and detection technologies. Key research gaps include limited data on rare earth metal toxicity and the effects of chronic low‐dose exposures. This study underscores the need for integrated global efforts in detecting, remediating, and mitigating risks.

## 1. Introduction

Toxic metallic effluents from industries present a significant threat to both the environment and human health. These effluents typically contain toxic heavy metals, which are by‐products of various industrial processes, including smelting, mining, electroplating, battery manufacturing, and chemical production [1]. When released into the environment, these metals do not degrade and can persist for long periods, accumulating in soil, water, and living organisms [2]. Beyond their environmental persistence, metallic pollutants enter biological systems through multiple exposure routes—primarily ingestion of contaminated food and water, inhalation of particulate‐bound metals, and dermal absorption. After internalization, these metals bind to and interact with essential biological components such as nucleic acids, proteins, and lipids often exerting leading to cellular and organ dysfunction [3]. These metallic pollutants are known to exert organ‐specific and cellular effects, targeting the liver, kidney, nervous system, and hematopoietic tissues.

The presence of toxic metals such as lead (Pb), mercury (Hg), cadmium (Cd), arsenic (As), titanium (Ti), scandium (Sc), beryllium (Be), aluminum (Al), thallium (Tl), chromium (Cr), and nickel (Ni) in industrial effluents raises serious environmental and public health concerns due to their persistence and potential for bioaccumulation. [4]. The harmful effects of these metals arise from their capacity to induce systemic metal toxicity by disrupting biological processes by inducing oxidative stress, inhibiting enzymes, and interfering with cellular functions in plants and animals [5]. High concentrations of heavy metals in effluents can lead to their biomagnification through the food chain, thereby increasing dietary heavy metal exposure and cumulative body burden, endangering human health through consuming contaminated water or aquatic life [6]. Metals exert toxicity through multiple mechanisms including excessive generation of reactive oxygen species (ROS), leading to oxidative stress, high affinity binding to thiol (–SH) moieties of cysteine residues in proteins and enzymes, resulting in irreversible enzyme inactivation [7], disruption of normal biochemical processes via molecular mimicking of essential metal cofactors, and modulation of the gene expression through epigenetic modifications and genetic mutation [8].

Traditional toxic metals such as Pb, Hg, Cd, and As continue to dominate toxicological research, emerging metals including aluminum, titanium, and scandium are increasingly associated due to their industrial development, nanoparticle applications, and lack of toxicological characterization. Their inclusion allows for a more comprehensive mapping of both known and emergent metallic contaminants, as well as the identification of unexplored research gaps.

Metals are typically categorized by their atomic weights or atomic numbers, and relatively high densities, although the specific criteria and inclusion of metalloids vary by context. Heavy metals are often defined by their density in metallurgical contexts, while in physics, the atomic number might be the distinguishing feature. Chemists, on the other hand, may prioritize chemical behavior [9]. Various specific definitions exist, yet none have gained universal acceptance. Among the definitions reviewed, up to 96 of the 118 known elements can be classified as heavy metals, but only mercury (Hg), lead (Pb), and bismuth (Bi) satisfy all criteria. Although there is no universally accepted definition, the term “heavy metal” is commonly used in scientific literature and discussions, often described as “a broad term that describes a group of naturally occurring metallic elements of high molecular weight and density compared to water” [10]. Not only heavy metals but also the elements present in the lower groups of the periodic table have adverse effects on both the environment and living organisms, but so far, research has shown that the toxicity emitted by heavy metals is much more dominant than the lower group metals [11]. Thus, while heavy metals are recognized as highly toxic and remain the dominant focus of environmental toxicology, the contribution of low‐density metals to environmental and health hazards cannot be overlooked [12]. Toxicity is ultimately governed not by an element’s density but by its dose, chemical reactivity, and biological interactions. Therefore, a comprehensive approach to metal toxicity research must encompass both heavy and low‐density metals to understand their cumulative and synergistic risks comprehensively [13].

Trace toxic metals can be categorized into essential and nonessential types. Essential trace toxic heavy metals are those required by organisms in small amounts for normal metabolic functions but can be toxic at higher concentrations [14]. Examples include iron (Fe), which is necessary for oxygen transport in the blood but can cause toxicity if accumulated excessively; zinc (Zn), which is vital for enzyme function and immune system performance, yet excessive amounts can disrupt cellular processes; and copper (Cu), which is essential for enzyme activity and nerve function, but toxic levels can cause liver and kidney damage [15]. Nonessential trace toxic heavy metals do not have any recognized biological function and can be harmful even at low concentrations. Examples include lead (Pb), mercury (Hg), and cadmium (Cd), aluminum (Al), and beryllium (Be), all of which lack beneficial biological roles and are associated with neurological, cardiovascular, and renal damage [16]. Table 1 summarizes the list of major toxic metals into essential and nonessential categories, highlighting their primary environmental sources, target organs, and toxicological effects.

**TABLE 1:** Essential and nonessential metals: environmental and biomedical relevance.

Metal	Essential/nonessential	Major sources	Primary toxic effects	Target organs	Environmental relevance	Biomedical relevance	References
Mercury (Hg)	Nonessential	Industrial discharge, mining, combustion, fish consumption	Severe neurotoxicity, oxidative stress, renal dysfunction	Brain, kidneys	Highly persistent, bioaccumulative in aquatic food chains	Developmental neurotoxicity; systemic toxicity	Ali et al. [17], El Ati‐Hellal et al. [18], Ehis‐Eriakha et al. [19], Hejna, M et al. [20], Abd Elnabi et al. [21]
Lead (Pb)	Nonessential	Industrial emissions, mining, contaminated water/soil	Neurotoxicity, encephalopathy, oxidative stress	Brain, kidneys, bones	Soil, air, and water contamination; biomagnification	Cognitive impairment; developmental toxicity	Ali et al. [17], El Ati‐Hellal et al. [18], Ehis‐Eriakha et al. [19], Hejna et al. [20], Abd Elnabi et al. [21]
Cadmium (Cd)	Nonessential	Industrial effluents, mining, dietary intake	Nephrotoxicity, oxidative stress, DNA damage	Kidneys, liver	Persistent, accumulates in soil and crops	Carcinogenic, renal dysfunction	Ali et al. [17], El Ati‐Hellal et al. [18], Ehis‐Eriakha et al. [19], Hejna et al. [20], Abd Elnabi et al. [21]
Arsenic (As)	Nonessential	Geological sources, industrial discharge	Carcinogenicity, oxidative stress	Skin, liver, kidneys	Groundwater contamination; long‐term persistence	Multiorgan toxicity; carcinogenic	Ali, H., et al. [17], Ehis‐Eriakha et al. [19], Hejna et al. [20], Abd Elnabi et al. [21]
Nickel (Ni)	Nonessential	Industrial activities, mining	Carcinogenicity, oxidative stress, DNA damage	Lung, liver, kidneys	Soil contamination; occupational exposure	Organ dysfunction; cancer risk	Saad et al. [22], Ehis‐Eriakha et al. [19], Abd Elnabi et al. [21]
Chromium (Cr)	Essential (Cr III)/Nonessential (Cr VI)	Industrial effluents, diet	Carcinogenicity oxidative stress, DNA damage	Lung, liver, kidneys	Industrial and occupational contamination	Glucose metabolism (Cr III); genotoxicity (Cr VI)	Saad et al. [22], Abd Elnabi et al. [21]
Aluminum (Al)	Nonessential	Environmental contamination, industrial use	Neurotoxicity	Brain	Widespread environmental occurrence	Possible link to neurodegeneration	Saad et al. [22],
Copper (Cu)	Essential	Diet, industrial activities	Oxidative stress, liver damage	Liver, kidneys	Soil contamination from effluents	Essential for enzymatic processes; toxic in excess	Saad et al. [22], Ehis‐Eriakha et al. [19], Hejna et al. [20]
Zinc (Zn)	Essential	Plants, animals, industrial discharge	Toxicity at high levels	Liver, kidneys	Soil contamination	Immune function; toxic in excess	Saad et al. [22], Ehis‐Eriakha et al. [19], Hejna et al. [20]
Manganese (Mn)	Essential	Nuts, cereals, industrial emissions	Neurological toxicity	Brain	Soil accumulation	Required for growth; neurotoxic at high levels	Saad et al. [22], Hejna et al. [20]
Cobalt (Co)	Essential	Soil, animal feed	Carcinogenic	Lung	Occupational exposure	Component of Vitamin B12	Saad et al. [22], Hejna et al. [20]
Selenium (Se)	Essential	Eggs, fish, cereals	Selenosis	Various organs	Soil variability; agricultural relevance	Antioxidant defense	Saad et al. [22], Hejna et al. [20]
Iron (Fe)	Essential	Biological fluids, animal feed	Hydroxyl radical formation	Blood, systemic organs	Soil contamination	Oxygen transport; oxidative stress in overload	Saad et al. [22], Hejna et al. [20]

**FIGURE 1:**
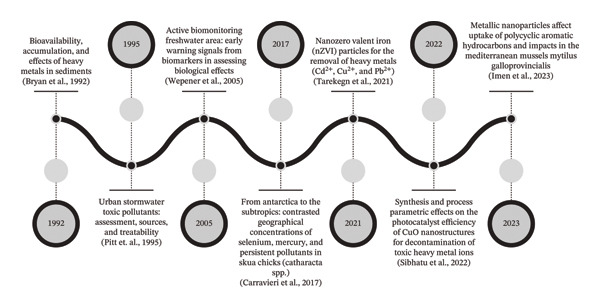
Overview of benchmarking milestones of metal toxicity research over the years.

Elevated levels of essential and nonessential trace elements may result in physiological disturbances mediated through metal‐induced oxidative stress and redox imbalance or genetic alterations indicative of metal genotoxicity and mutational instability [5]. The health hazards associated with heavy metal exposure depend on their concentration in a particular medium and the duration of exposure. Even minimal amounts of these harmful metals can lead to adverse health effects including cumulative metal genotoxicity under prolonged and chronic heavy metal exposure [3]. Figure 1 illustrates the key milestones in the evolution of metal toxicology research. From the early 1990s until 1995, research was mainly focused on descriptive studies, concentrating on identifying sources of heavy metal contamination in industrial effluents and highlighting environmental monitoring, concentration thresholds, and case‐based evidence of poisoning. By the early 2000s (around 2005), improvements in analytical chemistry and advances in cellular biology allowed for the investigation of bioaccumulation patterns and biomagnification across trophic levels. The field then transitioned toward mechanistic toxicology between 2010 and 2017, focusing on metal‐induced oxidative stress, enzyme inhibition, mitochondrial dysfunction, apoptosis, and genotoxic pathways at molecular and cellular levels. In more recent years (2021–2023), studies have incorporated toxicokinetic dose–response modeling, environmental risk assessment and public health policy issues into risk‐based and regulatory frameworks. Systems toxicology, omics technologies, computational modeling, and evaluations of nanometal interactions are now included in contemporary studies, representing a move away from merely observational toxicology and toward predictive, mechanistic, and policy‐oriented methodologies. This shift in time highlights the increasing complexity of metal toxicity research and supports the necessity for a thorough scientometric evaluation to map its thematic and intellectual development.

The scientometric analysis of heavy metal toxicity originating from industrial effluents involves an extensive and systematic assessment of research outputs and scientific literature in this field, utilizing advanced quantitative techniques [24]. This approach encompasses using bibliometric tools to evaluate trends, patterns, and the impact of scholarly publications, citations, and research activities related to the toxicity of metals discharged from industrial processes [25]. It seeks to deliver a comprehensive and systematic understanding of scientific progress, collaborations, and the dissemination of knowledge in this area, thereby supporting informed decision‐making and guiding future research directions [26].

Bibliometric analysis quantitatively assesses research outputs through publication counts, citation indices, and other statistical measures to delineate research trends, identify highly cited papers, and recognize influential contributors. Scientometric analysis expands upon this by examining the field’s broader scientific impact and evolution, including the study of research collaboration networks, technological innovations, and the dissemination pathways of scientific knowledge. Temporal analysis of publication trends provides insights into fluctuations in research activity, while citation analysis offers a deeper understanding of the reach and influence of specific studies and researchers. This integrative approach, leveraging scientometric methodologies, is instrumental in identifying critical research gaps, spotlighting leading researchers and institutions, and shaping future research agendas to report the deleterious heavy metal contamination effects from industrial effluents on public health and the environment [27].

## 2. Objectives

The primary objective of this study was to conduct a systematic mapping of the evolution of research concerning the metal toxicity and their harmful effects, with an emphasis on tracking the development of key research themes through networks of keywords. Despite an enormous amount of primary research and review articles dedicated to this field, existing analyses have predominantly focused on high‐density metals (“heavy metals”). To the best of our knowledge, a comprehensive scientometric analysis that integrates the study of both high‐density and low‐density metals has not been conducted, leaving a significant gap in our understanding of the field’s overall intellectual structure and evolution. This work aims to bridge this critical gap, providing a holistic and quantitative overview of metal toxicity research by analyzing the publication trends, key contributors, funding agencies, journals, and thematic evolution for both high‐density and low‐density metals. Moreover, this scientometric study is a valuable resource for researchers, funders, and policymakers to navigate the past, present, and future directions of metallic pollutant research.

### 2.1. Bibliometric Analysis

Bibliometric analysis necessitates large datasets with an adequate number of records and a sufficiently broad time span. It relies on various metrics, including the number of publications, citation counts, and cocitation frequencies. These metrics can be dissected by country, institution, and individual authors, as well as by examining the interconnections among these entities. This analytical approach is highly practical for understanding historical and emerging research trends within academic journals, enabling a comprehensive mapping of past progress and future directions. Researchers can investigate specific topics or research domains over designated periods by applying bibliometric analysis. This involves thoroughly examining all relevant journals according to pre‐established criteria [28]. The method allows for a detailed exploration of how research evolves, identifies key contributors and institutions, and uncovers research focus and output patterns. Such analysis is instrumental for tracking advancements, pinpointing gaps, and forecasting future trends within the academic literature.

## 3. Methodology

**FIGURE 2:**
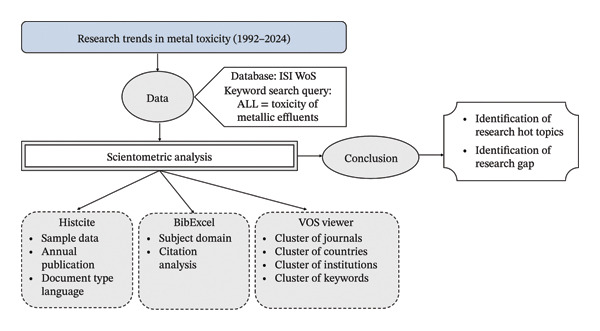
Framework of the research design for the current study.

The schematic framework illustrating the research design for this analytical study addressing metal toxicity is meticulously depicted in Figure 2. This framework encompasses a comprehensive outline of the methodological approach, incorporating various analytical dimensions and investigative stages relevant to the systematic evaluation of the reviewed literature and empirical evidence concerning the impacts and management of toxic metallic discharges [29]. The scientometric data about the literature on toxic metal pollutants, spanning from January 1992 to June 2024, have been meticulously extracted from the comprehensive database known as the ISI Web of Science (WoS), which provides access to a wide range of scientific literature studies across various disciplines. Managed by Clarivate Analytics, it indexes journals, conference proceedings, books, and patents, offering tools to track citations and analyze research trends [30]. Researchers use WoS to find high‐quality sources, measure the impact of their work, and identify influential papers and authors in their fields. This extraction process involved a thorough search and retrieval of relevant academic publications, including journal articles and conference papers that address the various aspects of toxic metal pollutants.

The keyword search term “ALL = Toxicity of metallic pollutants” was used in the advanced search mode of the WoS core collection to retrieve the data. The software underwent an extensive and rigorous evaluation process to ascertain the precision and reliability of the data it extracted. This scrupulous assessment was undertaken to verify that the data extraction protocols operated with exceptional accuracy and integrity. Notably, the evaluation was conducted without any additional screening procedures or filters, ensuring that the evaluation focused solely on the inherent accuracy and validity of the extracted data [31]. After data retrieval, all records were exported in CSV format and subsequently imported into required software (VOSviewer, BibExcel) for further processing. Duplicate entries were identified through cross‐checking DOI numbers, article titles, author names, and publication year. In cases where DOI information was unavailable, title‐based and metadata‐based comparisons were employed to ensure accuracy. All identified duplicates were removed prior to analysis to minimize bias and maintain the integrity of the scientometric results.

The scientometric analysis was conducted using specialized software tools, namely, HistCite, BibExcel, and VOSviewer, to process the documents extracted and converted into plain text. These software applications were employed to analyze and interpret the data derived from the plain text files, facilitating the evaluation of bibliometric and citation patterns. Each tool performed distinct functions [32]. HistCite helps analyze annual publications, document types, and citation patterns, providing insights into metal toxicity research’s intellectual structure and historical development. BibExcel enables efficient extraction and organization of bibliographic data, supporting subject domain mapping and in‐depth citation analysis. VOSviewer creates bibliometric maps of journals, countries, institutions, and keywords, allowing the visualization of collaborations, influential works, and emerging research themes. Together, these tools strengthen scientometric analysis by integrating quantitative data with visual exploration, ultimately helping to identify research hotspots and gaps in the field [33]. Additionally, VOSviewer aids in keyword analysis by highlighting frequently co‐occurring terms in research articles, uncovering trends and emerging topics. It also maps relationships between journals and publishers, offering insights into the academic publishing landscape. With its interactive visualizations, users can explore and zoom into specific parts of the maps to analyze complex data more effectively. Bibliometric terms are particular concepts and metrics used to analyze and assess scientific research based on bibliographic data [34]. The citation refers to a source of information or ideas used in a research work, providing details that allow readers to locate and verify the original material. The total number of times other works have cited a publication is referred to as the citation count. This metric is often used to gauge the influence or impact of a research paper or author. The impact factor (IF) is a metric that indicates the average number of citations received per year by recent articles published in a journal. It is widely used to evaluate the relative significance of a journal within its specific field. Coauthorship shows the practice of multiple researchers working together on a single publication. Analysis of coauthorship patterns can reveal collaboration networks and research partnerships. Cocitation occurs when other publications cite two documents together. This can indicate a thematic or conceptual relationship between the cited works.

## 4. Results

### 4.1. Basic Data of the Extracted Document for Scientometric Analysis

A thorough analysis performed using VOSviewer in July 2024 identified 220 publications across various document types, which are included in the current study. The dataset spans 32 years, from January 1992 to June 2024, covering significant research on the toxicity of metallic pollutants. Approximately 108 authors made contributions to this body of work, and the research findings were disseminated across 59 distinct journals. The study incorporated 77 unique keywords, facilitating a comprehensive understanding of the thematic focus, and the documents were published in six different languages. Additionally, 53 institutions participated in producing these scholarly works, underscoring the broad institutional collaboration involved in this research area. This detailed bibliometric analysis highlights the multidimensional contributions to the field, reflecting the global and interdisciplinary nature of research on metallic effluent toxicity [35].

### 4.2. Types of Documents and Languages of Publication

This section of the article discusses the document types and languages of publication relevant to the current study. According to the VOSviewer database, distinct document types have been identified. Among these, research articles are predominant in the communication on metallic pollutant toxicity, accounting for 183 documents or 83.2% of the total. Specifically, 166 are classified as research articles, representing 75.4%, while 14 are proceedings papers, comprising 6.4%. The remaining documents fall under the category of early access, with three papers, representing 1.3% of the total. Next is the review article (*n* = 37), constituting 16.8% of the document types.

**FIGURE 3:**
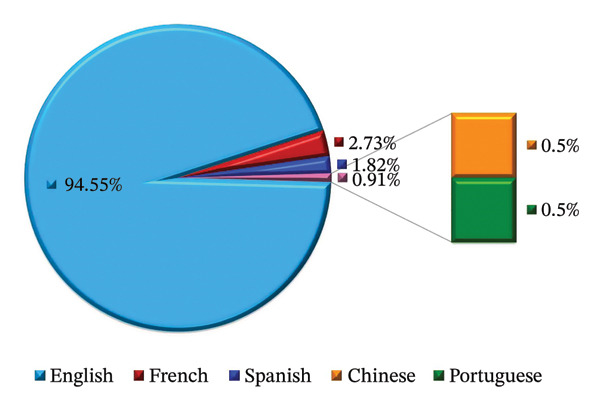
Number of published documents on metallic pollutants toxicity (1992–2024) classified by publication language.

Regarding impact, research articles surpass review articles in the number of publications and citation counts. The combined citation total for research and review articles is 8,719, with research articles contributing 5551 citations and review articles contributing 3168 citations. The toxicity of metallic pollutants is covered across 220 documents published in six languages. English is the primary language, with 208 papers (94.5% of the total), followed by French (2.7%), Spanish (1.8%), Chinese (0.5%), and Portuguese (0.5%) (Figure 3). Given the higher number of English‐language articles, this category also has the highest citation count of 8659.

### 4.3. Annual Trends of Publications

The distribution of research on this topic over the years plays a crucial role in fostering interest among young researchers. Emerging scholars can gain valuable insights into its trajectory by analyzing the chronological progression and evolutionary development of research efforts in this field. This understanding highlights key milestones and emphasizes areas of ongoing innovation, ultimately motivating new researchers to contribute to the field’s further advancement. The two distinguished phases are observed in Figures 4(a) and 4(b).

FIGURE 4(a) Number of published documents from 1992 to 2024 and (b) cumulative count of documents showing the exponential growth pattern.

**Figure:**
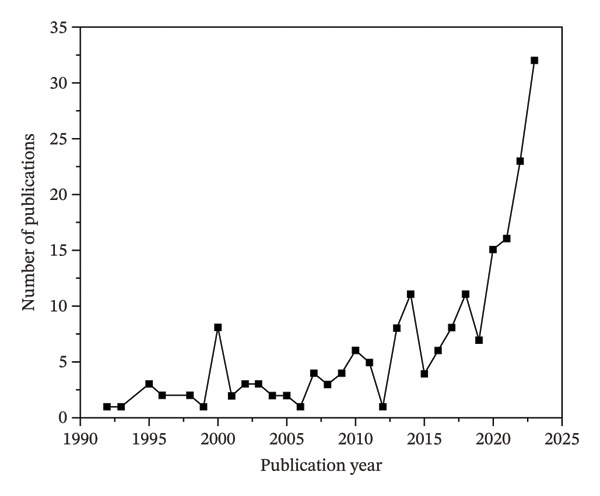
(a)

**Figure:**
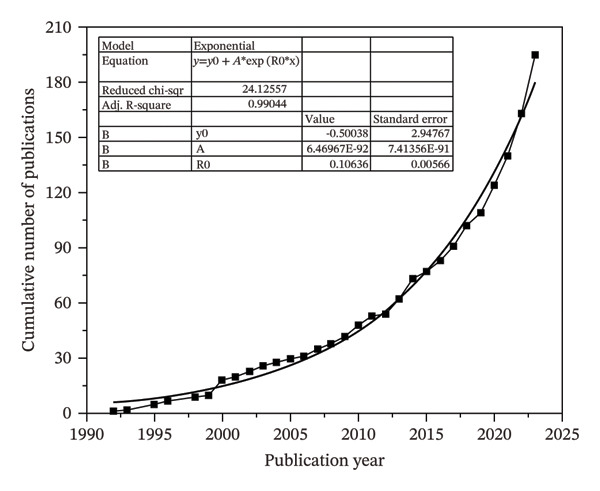
(b)

#### 4.3.1. Formative Phase (1992–2017)

This stage typically represents a period of slow growth, characterized by an inconsistent rise in the number of annual publications. Further segmentation of the formative phase, based on the cumulative volume of publications, reveals that before 1999, the total number of documents did not surpass 10. Starting from the 21^st^ century, however, the cumulative count began to show a gradual upward trend, with a steady increase of approximately 3–4 publications per year.

#### 4.3.2. Exponential Phase (2018–2024)

The cumulative number of publications reached 102 by the year 2018. The publication rate experienced a consistent rise after 2018, with more than 15 documents being published annually. Notably, 2023 recorded the highest number of publications in a single year, with 32 papers, and a total global citation score (TGCS) of 14.5%. An exponential growth trend was observed in this field, with projections indicating a continued increase in the coming years. Figure 4(b) depicts this trend based on the exponential function $y=y_{o}+A_{1}e_{o}^{\left(x-x\right)/t_{1}}$, where *y* represents the cumulative number of publications and *x* corresponds to the year.

### 4.4. Category Analysis

**FIGURE 5:**
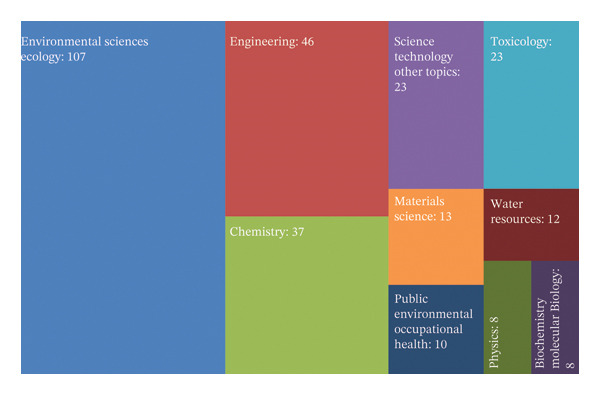
Top 10 Web of Science (WoS) subject categories related to metallic effluent toxicity research.

This bibliometric study offers a detailed analysis of the subject categories shown in Figure 5, encompassed by published research on the toxic metals. This evaluation provides researchers with a subject‐oriented perspective, facilitating targeted investigations within the field. Additionally, it demonstrates the comprehensive scope of the current study across a wide array of categories. The BibExcel analysis identifies both the most and least emphasized subject areas used in research conducted on this specific topic. Upon examining the distribution of subject categories, 39 distinct subject categories were identified for the present study. Based on the citation frequency and the number of publications, Environmental Science and Ecology emerged as the most frequently explored research domain, encompassing 107 publications (48.63% of the total output). This trend highlights the increasing attention given to emerging disciplines such as engineering, chemistry, science and technology, toxicology, and materials science.

In addition to subject areas directly related to toxicity, other categories such as Water Resources, Public Environmental and Occupational Health, Physics, and Biochemistry and Molecular Biology offer an integrated perspective, incorporating clinical and toxicological aspects of certain periodic elements and their nanoparticles. This diversity of sources contributes to a more holistic understanding of the topic.

### 4.5. Journal Analysis

Journal analysis underscores the critical importance of theme selection for future research endeavors. A total of 59 distinct publications have addressed the issue of toxic metals. Among these, the journal *Environmental Science and Pollution Research* stands out with the highest volume of publications (13). Following this, *The Science of the Total Environment* holds the second position with 12 publications. The *Chemical Engineering Journal*, *Environmental Pollution*, and *Separation and Purification Technology* occupy the subsequent ranks with publication shares of 3.6%, 2.7%, and 2.7%, respectively. In terms of citation metrics, *Environmental Pollution* leads with the highest citation count, encompassing 1222 citations. To analyze the interrelationships between journals, citation and bibliographic analyses were conducted using VOSviewer software. This software facilitated a comprehensive examination of citation patterns and bibliometric connections, providing insights into the strength of relationships among various journals within the field.

The IF is widely considered a critical metric that reflects an author’s academic reputation and the overall quality of their research. It functions as a key metric for evaluating the visibility, influence, and scholarly value of research articles and journals. A higher IF often indicates that a paper is frequently cited and has a significant influence in its field. In the context of the current study, it was observed that the journal *Environmental Pollution* has the highest journal IF (JIF), standing at 8.3. This positions it as one of the leading journals in environmental research, showcasing its high citation frequency and the importance of the studies it publishes. On the other hand, the journal *Environmental Sciences* follows closely in the second place, with a JIF of 7.6, further establishing its strong reputation in the scientific community for publishing impactful and high‐quality research. The top 10 journals with the highest total number of citation counts related to the toxicity of metallic pollutants were identified and mentioned in Table 2.

**TABLE 2:** Top 10 journals with the highest number of total citations on the toxicity of metallic pollutants.

Journal	TP	TC	CPP	IF	Subject category
Environmental Pollution	6	1222	203.6	7.6	Environmental Science and Ecology
Botanical Review	1	385	385	2.8	Plant Sciences
Science of the Total Environment	12	285	23.7	8.2	Science Technology
Journal of Nanomaterials	2	241	120.5	1	Science, Technology, Material Science
Water Environment Research	2	212	106	2.5	Water Resources, Environmental Science
Applied Catalysis B‐Environmental	2	170	85	20.2	Toxicology, Environmental Science
Current Medicinal Chemistry	1	155	155	3.5	Chemistry, Pharmacology Pharmacy
ACS Sustainable Chemistry and Engineering	1	142	142	7.1	Chemistry, Engineering
Environmental Science and Pollution Research	13	137	10.5	1.9	Environmental Science, Science Technology
Biosensors and Bioelectronics	1	132	132	10.7	Physics, Biophysics, Engineering

*Note:* TC—total citations received by the documents till the date of data retrieval, IF—impact factor of the journal 2024.

Abbreviations: CPP, citations per publication (TC/TP); TP, total publications.

### 4.6. Country Analysis

A comprehensive analysis of research output across various global regions, focusing on metal toxicity, highlights notable contributions from researchers worldwide. In total, 220 research papers were authored by 108 researchers representing 56 different countries. A bibliometric network analysis was conducted to evaluate countries’ research productivity, using key criteria such as the total number of publications and the number of citations received. The top 15 countries and institutes were identified and are mentioned in Table 3 based on the number of publications. In terms of total publications, China leads the global ranking, contributing 58 publications, which represent 26.4% of the total output. France follows in second place, with 38 publications, accounting for 17.3%. India ranks third, with 19 publications, comprising 8.6% of the overall research output. The United States and Brazil rank fourth and fifth, with 17 and 15 publications, respectively. When shifting the focus to citation‐based analysis, the United Kingdom had the highest number of citations, with a total of 2395 citations for its research papers. China ranks second in this category, with a total of 1355 citations. India, ranks third, with 1144 total citations for its contributions. France and the United States occupy the fourth and fifth positions, respectively. This analysis highlights the leading countries in the field of metal toxicity research, showcasing not only their volume of publications but also the impact and recognition their research has garnered globally.

**TABLE 3:** Top 15 countries and institutes based on the highest number of publications on the toxicity of metallic pollutants.

Country	TP	TC	CPP	Institution	TP	TC	CPP
China	58	1355	23.4	Chinese Acad Sci	10	272	27.2
France	38	912	24	Univ Bordeaux	6	135	22.5
India	19	1144	60.2	Univ Chinese Acad Sci	5	69	13.8
USA	17	822	48.3	East China Univ Sci and Technol	4	183	45.7
Brazil	15	146	9.7	Sichuan Normal Univ	4	5	1.25
Spain	11	436	39.6	Univ Aveiro	4	98	24.5
Pakistan	10	333	33.3	Univ Carthage	4	42	10.5
Malaysia	8	119	14.9	US EPA	4	391	97.7
Italy	7	290	41.4	Aix Marseille Univ	3	108	36
Morocco	7	185	26.4	Chinese Acad Agr Sci	3	34	11.3
Poland	6	113	18.8	Govt Coll Univ	3	65	21.7
Portugal	6	152	25.3	Hanyang Univ	3	167	55.7
South Korea	6	196	32.7	Harbin Inst Technol	3	53	17.7
Tunisia	6	95	15.8	Huazhong Univ Sci and Technol	3	10	3.3
Iran	5	116	23.2	Jagiellonian Univ	3	75	25

*Note:* TC—total citations received by the documents till the date of data retrieval.

Abbreviations: CPP, citations per publication (TC/TP); TP, total publications.

Collaboration in coauthorship enhances the credibility and recognition of the review within the academic community, as multiple well‐regarded researchers contribute their insights (Figure 6(a)). Coauthorship in articles on metal toxicity revealed interesting global trends, particularly in China, which is leading in the highest number of citations, demonstrating its prominent contribution to this field. Chinese researchers have consistently published influential work, reflecting their significant role in advancing knowledge on metallic pollutants’ environmental and biological impacts. Their collaboration networks are vast, which has increased their research’s global visibility and impact. On the other hand, Saudi Arabia has shown a promising trend in increasing coauthorship links and publications on toxic metals. Researchers in Saudi Arabia are progressively building stronger collaborative ties with international researchers, enhancing the quantity and quality of articles produced. This growth in coauthorship, particularly in environmental sciences, signals a strategic focus on tackling regional pollution issues while contributing to the global body of knowledge [36]. The combined efforts of China and Saudi Arabia underscore the importance of international collaboration in addressing the complex challenges posed by toxic metals, improving both research productivity and the global reach of scientific findings. The frequency of research with each country is depicted in Figure 6(b).

FIGURE 6(a) Countrywise coauthorship analysis using VOSviewer. Minimum threshold: 5, total countries: 56, countries meet the threshold: 17, total link strength: 126. (b) Geographic distribution of research output and collaborative networks between the countries. The larger the country’s productivity, the deeper the color it possesses.

**Figure:**
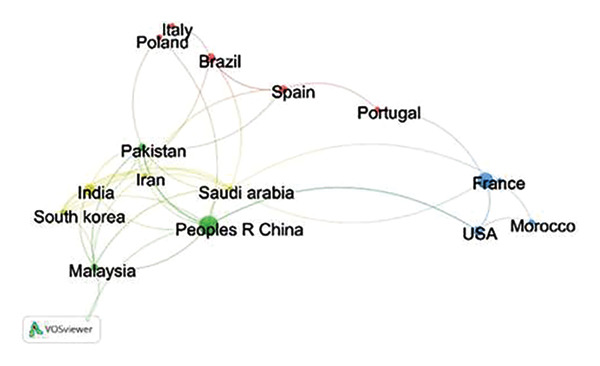
(a)

**Figure:**
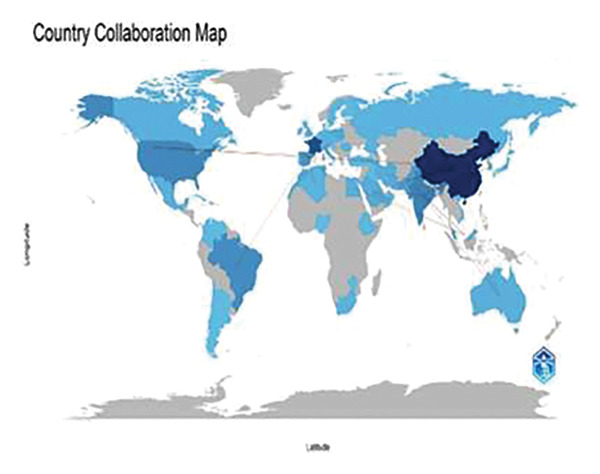
(b)

### 4.7. Institutional Analysis

According to the BibExcel analysis, 53 universities, institutions, and research organizations have contributed to the body of work encompassing not only the general concept of metallic toxicity but also the toxicity associated with specific metallic elements and the release of toxic metals from various industries and other sources. From this current study, the Chinese Academy of Sciences leads in the number of publications connected to our research topic, surpassing other institutions. The University of Bordeaux ranks second, with a total of six publications. Among these contributors, India has made a significant contribution with 19 publications. This includes research from prominent institutions such as the University of Calcutta and the Council of Scientific and Industrial Research (CSIR), each contributing 0.9% of the total publications. Additionally, certain Indian universities collectively contribute 1.4% of the publications.

Conversely, numerous academic institutions from Morocco, Canada, and Portugal, such as the University of Cadi Ayyad, the University of Calgary, and the University of Coimbra, respectively, have demonstrated a promising trajectory toward expanding their research efforts in this domain in the coming years. These universities are poised to significantly enhance their contributions to the field of heavy metal toxicity from industrial effluents, with anticipated growth in both the volume and impact of their scholarly output [37]. Their strategic focus on advancing research in this area indicates a commitment to addressing critical environmental and public health challenges associated with industrial pollutants, positioning these institutions as key players in driving future scientific innovation and discovery [38].

### 4.8. Most Cited Articles

Citation is crucial as it recognizes the original authors and supports the credibility of the information presented. It allows readers to trace the source of the ideas, research, or data, ensuring transparency and fostering intellectual integrity [39]. Citing authoritative, peer‐reviewed articles enhances the reliability of the review and helps position the work within the broader scientific context. Highlighting the most cited articles on a particular topic adds value by pointing readers to influential studies that have shaped the field. These highly cited articles often represent foundational or groundbreaking research, the basis for ongoing scientific advancements and discussions.

A comprehensive list of the top 10 most cited articles is provided in Table 4. The top five most cited articles on the toxic metals are discussed below, highlighting their key findings and impact on the field.

**TABLE 4:** Top 10 most cited articles on the toxicity of metallic pollutants (1992–2024).

Citation rank	Article title	Publication year	TC	Ref.
1	Metals, minerals and microbes: geomicrobiology and bioremediation	2010	1236	[40]
2	Bioavailability, accumulation and effects of heavy metals in sediments with special reference to U.K. estuaries: A review	1992	1128	[41]
3	Comparison of mercury, lead and arsenic with respect to genotoxic effects on plant systems and the development of genetic tolerance	2004	453	[42]
4	Mercury toxicity in plants	2000	385	[43]
5	Urban stormwater toxic pollutants: assessment, sources, and treatability	1995	211	[44]
6	Synthesis approaches of zinc oxide nanoparticles: the dilemma of ecotoxicity	2017	190	[45]
7	N‐Doped porous carbon with magnetic particles formed in situ for enhanced Cr (VI) removal	2013	186	[46]
8	Toxicity of nanoparticles	2014	155	[47]
9	Comparative effects of cadmium, copper, paraquat and benzo [a] pyrene on the actin cytoskeleton and production of reactive oxygen species (ROS) in mussel hemocytes	2003	151	[48]
10	Anodic oxidation for the degradation of organic pollutants: anode materials, operating conditions and mechanisms	2021	149	[49]

*Note:* TC—total citations received by the documents till the date of data retrieval.

#### 4.8.1. Metals, Minerals, and Microbes: Geomicrobiology and Bioremediation

The article authored by Gadd et al., published in 2010, holds the top position in terms of citation count within the subject category of Microbiology. This article provides a comprehensive overview of the interactions between micro‐organisms and metallic minerals within the biosphere, focusing on biotransformation and biogeochemical cycling processes. The study delves into the intricate relationships between microbial communities and metallic compounds, examining how micro‐organisms influence metals’ transformation, mobility, and fate within environmental systems. Additionally, it explores the critical role of biological systems in the remediation of metal and radionuclide contaminants through bioremediation and detoxification. This involves leveraging microbial metabolism to neutralize or eliminate toxic metals and radioactive pollutants from ecosystems. Addressing these complex biochemical interactions, the article highlights the potential of utilizing microbial processes for environmental clean‐up, particularly in mitigating the harmful effects of metallic and radionuclide pollutants. The work is a key reference in ecological microbiology, contributing to the theoretical understanding and practical applications of microbial interactions with metallic contaminants.

#### 4.8.2. Bioavailability, Accumulation, and Effects of Heavy Metals in Sediments With Special Reference to U.K. Estuaries: A Review

The review article, authored by Bryan et al. and published in 1991, ranks second in terms of total citations within its field. This study is region‐specific, concentrating on the estuarine ecosystems of the United Kingdom. The paper provides a detailed analysis of key processes related to the bioavailability and accumulation of heavy metals in estuarine sediments. The significant focus of this review is on how these accumulated metals can undergo biogeochemical transformations into organometallic compounds, which are known to exhibit enhanced bioavailability and elevated toxicity levels. Moreover, the paper addresses the future outlook for maintaining the sustainability of avian fauna, which thrives in estuarine ecosystems and could be severely impacted by heavy metal contamination. The authors emphasize the importance of ongoing conservation efforts to protect these bird populations, which are integral to the health and stability of estuarine ecosystems. Overall, the article is a critical resource for understanding the environmental dynamics of heavy metal pollution in U.K. estuaries, highlighting both the immediate risks of metal accumulation and the long‐term implications for wildlife, particularly avian species.

#### 4.8.3. Comparison of Mercury, Lead, and Arsenic With Respect to Genotoxic Effects on Plant Systems and the Development of Genetic Tolerance

The paper, authored by M. Patra et al. from the University of Calcutta and published in 2004 in Environmental and Experimental Botany, presents an element‐specific study on metal toxicity. This research provides a wide‐ranging examination of the toxicological impacts of various metals on plants, alongside the genetic mechanisms plants undertake to mitigate these harmful effects. The study highlights the integration of metal‐induced stress responses in plants. It offers detailed insights into how plants adapt genetically to tolerate high levels of toxic metals, thereby reducing potential damage. It delves into the physiological and metabolic pathways affected by metal toxicity, making it a valuable resource for understanding the molecular basis of plant responses to toxic metal exposure. The key focus of the paper is on arsenite detoxification strategies. The authors provide an in‐depth exploration of the various detoxification mechanisms employed by plants to manage arsenite toxicity, including chelation, sequestration, and oxidation–reduction reactions. These processes are crucial for maintaining plant metabolism in contaminated environments and enhancing plant resilience against metal stress. This research serves as a complete reference for understanding the integration of toxic metals into plant metabolic pathways and the genetic tolerance mechanisms that allow plants to survive and thrive in metal‐contaminated environments. The paper contributes significantly to environmental botany by elucidating the complex interactions between plants and toxic metals, with a particular emphasis on arsenite detoxification methodologies.

#### 4.8.4. Mercury Toxicity in Plants

This botanical review, published in 2000, focuses on mercury toxicity in plants and ranks fourth in total citations. The paper is based on a comprehensive set of experimental studies investigating mercury compounds’ genetic effects across various genetic endpoints. These experiments provide crucial insights into how mercury, as a toxic metal, impacts plant genetic systems. The review elucidates the mechanisms through which poisonous metals, such as mercury, penetrate plant cells. It explains that mercury enters the cell through pathways analogous to those used to uptake essential micronutrient metal ions, exploiting the plants’ natural nutrient acquisition processes. This provides a clear understanding of how plants can inadvertently absorb toxic metals due to similarities in their ionic structure with essential elements. Furthermore, the paper advocates the implementation of robust and standardized phytoremediation techniques as an effective strategy to detoxify and remove heavy metals, including mercury, from contaminated soils and surrounding environments. In summary, this review serves as a valuable resource for understanding the genetic and cellular impact of mercury toxicity in plants, while also proposing phytoremediation as a sustainable and efficient approach for the remediation of mercury‐contaminated ecosystems.

#### 4.8.5. Urban Stormwater Toxic Pollutants Assessment, Sources, and Treatability

The research article by Pitt et al., published in 1995 in Water Environmental Research, explores pollutants and toxic substances in urban stormwater, beyond the typically high levels of pollution caused by industrial contaminants. The study employs a variety of purity assessments, toxicity screening assays, and other analytical techniques to detect even trace amounts of toxic components present in urban stormwater, which often exhibit elevated concentrations of harmful substances. The paper emphasizes the need for comprehensive studies of biological community structures to understand better the specific impacts of these pollutants on natural ecosystems, including water sources and plant life. Furthermore, it highlights the importance of identifying and developing effective methods and processes for removing these toxic pollutants, regardless of their origin or nature.

### 4.9. Funding Agencies

Incorporating funding agency details into a review article is essential, as it acknowledges the organizations supporting research, ensures transparency regarding financial assistance, and identifies potential influences on the scientific direction. Credibility is increased by funding from respectable national science foundations and international organizations, as these grants usually undergo thorough review and are in line with established international agendas. Based on the analysis from 1992 to 2024 (Figures 7(a) and 7(b)), the National Natural Science Foundation of China (NSFC) emerges as the dominant funding body, contributing to the highest number of publications (41) and accounting for the most significant citation impact (1471). This indicates that NSFC has prioritized research on toxic metals with a strong emphasis on human health and environmental risk assessments, which remain the core research domain of global concern.

FIGURE 7Funding impact on toxic metal research: (a) publications, (b) citations count, and thematic distribution of funding agencies (1992–2024).

**Figure:**
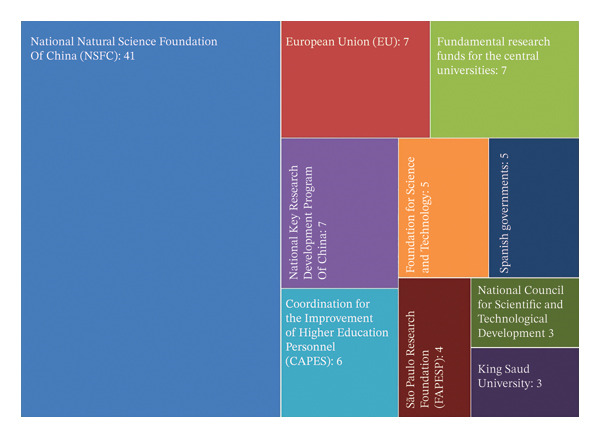
(a)

**Figure:**
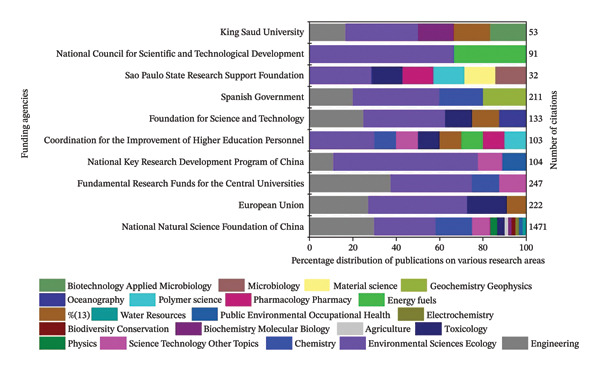
(b)

On the other hand, the European Union (EU) and the Fundamental Research Funds for the Central Universities had comparatively high citation counts (247 and 222, respectively), even though they contributed fewer papers. These trends infer that their funding has been deliberately allocated to practical research fields, specifically remediation and bioremediation techniques, which reflects a global emphasis on environmentally friendly pollution control. Similarly, the National Key Research and Development Program of China (211 citations) has been dynamic in advancing fundamental research, supporting mechanistic studies of cellular and molecular toxicity, pollutant transport, and ecosystem‐level impacts. However, organizations such as King Saud University (53 citations) and the São Paulo Research Foundation (32 citations) have funded more regional studies, frequently tackling new areas of study, including region‐specific contamination and less‐studied metals (e.g., Ga, In, Sn, and Sb). The Coordination for the Improvement of Higher Education Personnel (CAPES, Brazil) has frequently supported only a few articles with 103 citations to improve bioremediation research and capacity building in Latin America.

The Spanish Government (211 citations) has a comparatively minor publication output but a high citation impact, reflecting its focus on environmental monitoring and remediation in Mediterranean ecosystems, which are vulnerable to heavy metal contamination. With minimum publications, the Foundation for Science and Technology, Portugal (133 citations), has also promoted research in ecotoxicology and sustainable remediation technologies, indicating regional specialization within Europe. With significantly fewer publications, the National Council for Scientific and Technological Development, Brazil (91 citations), has emphasized toxicological risk assessments in tropical ecosystems, addressing geographic gaps often overlooked in global research. Finally, with minimal publications, King Saud University (53 citations) has contributed to emerging studies on nanoparticle–metal interactions and localized monitoring of pollutants, reflecting the increasing research momentum in the Middle East. These agencies contribute significantly to the worldwide body of knowledge on metallic pollutant toxicity, handle region‐specific contamination issues, and diversify research views, even though their output is less than that of global leaders.

While funding agencies vary across countries, research investments often converge on globally significant themes. This study considered the thematic overlap, such as research on metal toxicity, as the basis for comparison, rather than the identity of the funding agencies. This approach allows the establishment of a baseline that extends beyond country‐specific funding structures.

### 4.10. Keyword Analysis

Keyword analysis is a research method used to identify, analyze, and evaluate the most relevant and frequently occurring terms within a given dataset, such as documents and academic literature. It is crucial in understanding trends, patterns, and key themes across various fields. The most frequently used terms indicate key research trends and thematic focus areas on the toxicity of metallic pollutants, which were analyzed and are presented in Figure 8(a). A knowledge map for keyword co‐occurrence was constructed, comprising 77 terms organized into distinct clusters. Among these, “toxicity” emerged as the most relevant keyword for the current research focus, with the highest occurrence frequency of 80. Table 4 illustrates the top 20 keywords, their respective occurrences, and total link strength. By categorizing these keywords into specific groups, researchers and readers can better understand the nuances involved in searching for research articles or review papers. This approach enhances the precision and relevance of keyword selection within the context of this study. Numerous studies suggest that metal toxicity is predominantly associated with heavy metals. Due to their significant role in environmental pollution and their detrimental effects on living organisms, heavy metals rank as the second most prevalent contaminants. To enhance the efficiency of document retrieval and streamline the research process, categorizing keywords based on specific aspects of metal toxicity is essential.

FIGURE 8(a) Author keyword analysis for research on the toxicity of metallic pollutants, (b) VOSviewer co‐occurrence network and overlay visualization by time of author keywords. Minimum threshold: 5, total keywords: 1782, keywords that meet the threshold: 77, total link strength: 3096.

**Figure:**
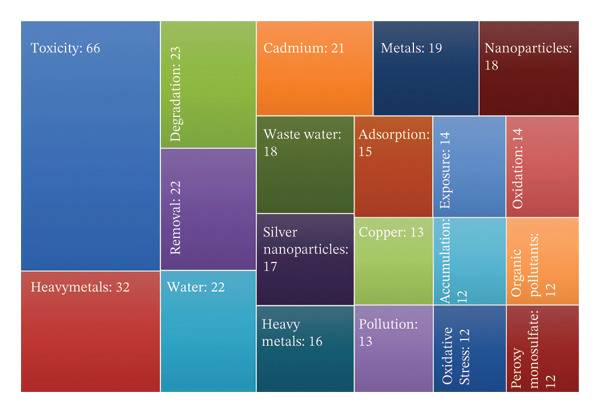
(a)

**Figure:**
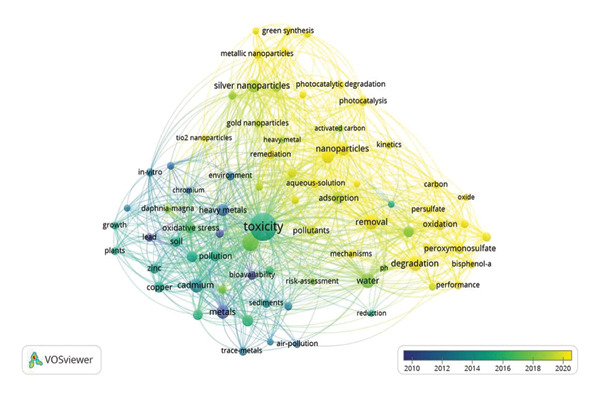
(b)

Keywords related to the environmental impact of toxic metals include “soil,” “wastewater,” and “sir pollution.” These terms facilitate targeted searches when investigating the ecological consequences of metal pollutants. While discussing the toxicity of metallic pollutants, there is a need for classifying the keywords based explicitly on individual metallic effects on the environment and on the organisms. “Cadmium,” “copper,” “mercury,” “persulfate,” “carbon,” “chromium,” and “graphene oxide” are the elemental specific keywords that were focused on in this study. Metals sometimes do not directly cause harmful effects to the environment. They are transformed into nanoparticles and create environmental instability. “Nanoparticles,” “silver nanoparticles,” “ZnO nanoparticles,” “nanomaterials,” and “gold nanoparticles” are keywords classified based on metallic nanoparticles and their harmfulness. From 1992 to 2024, many studies were carried out to find solutions to degrade or detoxify the toxic metals. For research focused on removal techniques and remediation strategies, relevant keywords such as “metal accumulation,” “bioremediation,” “bioaccumulation,” and “photocatalytic activity” are commonly employed. These keywords serve as precise search terms for identifying studies on effective methods for mitigating metal toxicity in various environmental matrices.

The co‐occurrence network visualization, generated using VOSviewer, reveals the clusters of interconnected keywords, reflecting the structure and evolution of research topics within the field, as depicted in Figure 8(b). By examining the frequency and co‐occurrence of keywords across multiple articles, researchers can obtain meaningful insights into the prevailing themes and emerging focus areas. Table 5 presents the top 20 author keywords related to research on the toxicity of metallic pollutants, determined by their frequency of appearance and total link strength. These keywords reflect the dominant themes and emerging trends in the field. This process helps to structure the literature, making it easier to recognize research gaps, highlight areas of consensus, and uncover potential avenues for future exploration. This, in turn, aids other researchers in locating relevant studies efficiently, facilitating a deeper understanding of the topic and promoting informed decision‐making in research design and strategy.

**TABLE 5:** List of top 20 author keywords related to the toxicity of metallic pollutants publications (1992–2024), ranked by total link strength and frequency of occurrence.

Keywords	Links	Total link strength	Occurrences
Toxicity	71	265	80
Heavy metals	49	109	32
Degradation	32	76	23
Removal	44	87	22
Water	37	82	22
Cadmium	37	78	21
Metals	29	63	19
Nanoparticles	47	84	18
Waste water	41	73	18
Silver nanoparticles	36	82	17
Heavy metals	34	55	16
Adsorption	44	68	15
Exposure	30	54	14
Oxidation	28	59	14
Copper	27	50	13
Pollution	29	45	13
Accumulation	30	47	12
Organic pollutants	32	46	12
Oxidative stress	31	56	12
Peroxymonosulfate	21	46	12

Overlay visualization of keyword co‐occurrence (Figure 8(b)) illustrates the timeline view of author keywords. The color scheme named viridis symbolizes the time‐varying keyword occurrences, which provides evolution from blue to green to yellow. Research on toxic metals prior to 2012 primarily addressed broad environmental concerns, with keywords emphasizing metals, heavy metals, air pollution, bioavailability, and specific toxic elements such as lead, cadmium, and chromium. This reflects a preliminary phase in which studies were largely focused on elemental toxicity and general environmental effects, and limited attention was given to mechanistic pathways such as metal‐induced oxidative stress. During the 2012–2018 period, research activity intensified, and additional mechanistic insights began to emerge. Keywords such as oxidative stress, reduction, in vitro studies, sediments, soil, plants, zinc, and copper indicate a growing emphasis on understanding biological impacts, environmental distribution, and organismal responses to heavy metal exposure and bioaccumulation. After 2018, the field underwent a significant shift toward advanced mitigation strategies and nanotechnology‐based approaches. This evolution is reflected in keywords such as removal, remediation, adsorption, photocatalysis, peroxymonosulfate, nanoparticles, TiO_2_ nanoparticles, gold and silver nanoparticles, and green synthesis. At the same time, ecological and toxicological assessments gained prominence, highlighted by terms such as risk assessments and *Daphnia magna*. A notable shift from basic toxicity studies to advanced remediation methods and risk‐based assessments is evident in this temporal keyword evolution. Table 6 lists the keywords of related publications that emerged during different time periods.

**TABLE 6:** Evolution of author keywords related to toxic metallic pollutants research during different time periods.

Period	Keywords
Before 2012	Metals, heavy metals, environment, stress, air pollution, bioavailability, lead, cadmium, chromium.
2012–2018	Trace‐metals, oxidative stress, reduction, in vitro, toxicity, sediments, growth, soil, plants, zinc, copper.
After 2018	Removal, remediation, degradation, mechanism, activated carbon, adsorption, photocatalysis, photocatalytic degradation, peroxymonosulfate, persulfate, bisphenol A, kinetics, green synthesis, nanoparticles, gold nanoparticles, silver nanoparticles, metallic nanoparticles, TiO_2_ nanoparticles, Daphnia magna, risk assessments.

## 5. Discussion and Future Perspectives

This scientometric analysis provides valuable insights for researchers interested in the emerging field of metal toxicity [50]. The article elaborates on key aspects, including the IF, the volume of publications, the journals in which these articles were published, the subject classifications, and the funding agencies that have actively supported high‐level research in this domain. In the process of gathering literature on the specific topic of metal toxicity, a total of 220 documents were collected, spanning the period from January 1992 to June 2024. Of these, 183 are original research articles, while 37 are review papers. Given that this is an evolving area of research, it is anticipated that in the coming years, an increasing number of researchers will actively engage in studies on metal toxicity and its detrimental effects on the environment [51].

The choice of publication language is instrumental in scientific research, enabling researchers to share their findings in a universally accessible format. English has emerged as the most widely accepted and spoken language globally, due to a convergence of historical, economic, and technological influences. Industries contributing to toxic metal pollution emerged in the 1960s–1970s with a focus on economic benefits, while the environmental and health risks only gained scientific attention in the late 1980s, leading to the first documented research in 1992 and a subsequent surge in related publications [52].

Category analysis sheds light on the research focus within specific disciplines. In the realm of metal toxicity and its environmental repercussions, there has been a pronounced shift in researchers’ attention toward the fields of environmental sciences and ecology. These domains have become focal points due to their relevance in understanding and mitigating the ecological consequences of toxic metal exposure. Biochemistry, molecular biology, and physics have significantly contributed to metal toxicity research by elucidating molecular mechanisms and explaining the physical interactions and dispersal of contaminants in environmental matrices. Moreover, the integration of metal toxicity research with public environmental health and occupational health is becoming more prominent, reflecting a deeper and more advanced interdisciplinary approach. This intersection emphasizes the importance of not only addressing the environmental consequences of metal pollutants, but also understanding their impacts on human health, particularly in occupational settings where exposure risks are elevated [53]. The growing focus on epidemiological studies and biomonitoring highlights the public health relevance of metallic pollutants, linking chronic exposure to neurotoxicity, nephrotoxicity, carcinogenicity, developmental toxicity, and endocrine disruption in human populations [54].

Chronic occupational exposure has been associated with alterations in biomarkers indicative of metal‐induced genotoxicity. The increasing prominence of biomarkers such as lipid peroxidation markers, antioxidant enzymes (SOD, CAT, GPx), metallothioneins, and inflammatory mediators in recent decades reflects a shift toward mechanistic and molecular toxicology approaches [55]. This convergence of fields illustrates the growing complexity and depth of scientific efforts aimed at addressing the multifaceted challenges of metal toxicity.

Journal analysis is more important because it ensures the credibility and validity of the research being reviewed. By critically evaluating journals, researchers can distinguish high‐quality, peer‐reviewed studies from irrelevant sources, which is essential for producing accurate and trustworthy conclusions. It also helps in understanding the latest advancements, identifying trends, and recognizing influential studies within a field. Moreover, it allows for a more comprehensive and balanced review by incorporating diverse perspectives, ultimately leading to a deeper and more enhanced understanding of the topic. Similar to the distribution across subject categories, a significant volume of articles has been published in the journal Environmental Sciences and Pollution Research. This journal has become a prominent platform for disseminating studies related to metal toxicity and environmental pollution. Additionally, articles published in Environmental Pollution have garnered a higher citation count, reflecting their substantial influence within the academic community. This journal not only leads in terms of citation impact but also surpasses others with the highest IF, underscoring its dominant position in the field of environmental research. These metrics highlight the journal’s critical role in advancing scientific understanding of pollution and its far‐reaching effects on ecosystems and public health.

Institutional analysis is more important because it helps assess the role and influence of institutions, such as governments and educational bodies, on societal, economic, and political outcomes. By examining how institutions operate, shape policies, and interact with other entities, researchers can better understand the frameworks that govern decision‐making, resource distribution, and power dynamics. A total of 56 different countries have contributed to the body of research on metal toxicity. Among these, China emerges as the dominant nation in the global landscape, accounting for 26.4% of all publications in this field. This substantial proportion reflects China’s significant investment and focuses on research related to metal toxicity. In terms of citation metrics, the United Kingdom holds the highest citation count for its published papers in this area. The relatively higher citation impact of the United Kingdom, despite a lower overall publication volume, suggests that its contributions to the subject have a strong influence. This may be qualified to the publication of foundational studies and comprehensive reviews in high‐impact journals, along with the U.K.’s early leadership in environmental regulatory toxicology and risk assessment frameworks. In contrast, China’s dominant position in publishing production reflects extensive research investment and rapid industrial development. However, many of its contributions are relatively recent and may not yet have achieved comparable citation density due to the citation maturity effects. Differences in journal visibility, worldwide collaboration networks, and research placement could help to explain the observed difference between publication volume and citation impact. The disparities in publication and citation rates among countries highlight the varying levels of engagement and expertise in the field of metal toxicity research on a global scale.

The Chinese Academy of Sciences stands out as the leading institution in terms of contributions to the research on metal toxicity, evidenced by its substantial number of publications. Moreover, institutions such as the University of Cadi Ayyad, the University of Coimbra, and the University of Calgary have demonstrated remarkable progress, underscoring their commitment to this domain. These institutions have played a pivotal role in fostering further investigations into metal toxicity and its implications, thereby enhancing the overall body of knowledge in this critical area of study. Their collaborative efforts and dedication to research underscore the importance of interdisciplinary approaches and international cooperation in addressing the challenges posed by metal toxicity and environmental pollution [56]. By examining data and research specific to individual countries, researchers can identify variations, trends, or patterns that may not be evident in broader, global studies. This analysis helps highlight differences in policy effectiveness, socioeconomic challenges, and institutional responses, making it possible to draw more accurate and nuanced conclusions. Additionally, country analysis ensures that the review considers diverse perspectives and regional contexts, leading to more comprehensive and applicable findings.

The importance of highlighting funding agencies in this paper cannot be overstated, as they have provided their indispensable contribution in supporting and sustaining scientific research. Securing adequate funding is a critical factor that allows researchers to conduct in‐depth investigations, acquire necessary resources, and collaborate across disciplines. By detailing the contributions of the major funding agencies, this study emphasizes how essential financial support is in driving impactful research. This distribution of emphasis across funding priorities demonstrates a structured global approach, such as core domains target urgent health risks, applied research advances technological solutions, fundamental research deepens mechanistic understanding, and emerging frontiers highlight future directions. Together, these findings reveal how funding not only drives publication productivity but also shapes the thematic evolution and impact of toxic metallic pollutant research. Importantly, these funding‐driven priorities also align with the United Nations Sustainable Development Goals (SDGs), the evolving research landscape reflects not only academic priorities but also a global commitment to sustainable environmental management and public health protection.

The extent to which an article is cited and its visibility in search results largely depends on the relevance and effectiveness of the keywords associated with the publication. In this case, the keywords are categorized into general and specific (nongeneral) terms. General keywords such as “toxicity,” “pollution,” and “heavy metals” are broad search terms that can be used to locate this scientometric analysis on metal toxicity. These terms cover the overarching themes of the study, making the article accessible to wider readers. On the other hand, specific keywords such as “ZnO,” “nanoparticles,” “mercury,” and “bioremediation” indicate a more precise focus within the article. These keywords highlight the particular toxic substances, such as zinc oxide (ZnO) and mercury, and the advanced methods utilized to mitigate their harmful effects, such as nanoparticles and bioremediation techniques [57]. The specificity of these terms reflects the article’s focus on both the harmful products resulting from metal toxicity and the innovative strategies employed to remediate and address these environmental challenges. By incorporating both general and specific keywords, the article enhances its discoverability and relevance within various branches of scientific inquiry related to environmental pollution and toxicology.

Beyond thematic clusters, scientometric signals also reflect pollutant‐specific mechanistic insights. For example, the prominence of cadmium research aligns with its well‐documented endocrine disruption and genotoxicity, reflecting its interference with hormonal pathways and DNA integrity. Likewise, lead is frequently linked to neurotoxicity particularly through calcium mimicry that disrupts synaptic signaling. Mercury emerges strongly in association with oxidative stress and neurodegeneration, reflecting its mechanistic role in ROS‐mediated neuronal damage [14, 58, 59]. These associations demonstrate how scientometric patterns not only capture research volume but also trace the mechanistic trajectories of individual metallic pollutants.

Integrating the mechanistic insights of toxic metal interactions in biological systems provides a comprehensive understanding of emerging research trends in metal toxicology.

The prominence of keywords “oxidation” and “oxidative stress” reflects the scientific attention toward ROS‐mediated cellular injury as a central pathway of metal toxicity. The appearance of “DNA damage” keyword further links these oxidative processes to genotoxic outcomes, including strand breaks and genomic instability. Keywords such as “activation” and “responses” highlight growing attention to molecular signaling pathways, including stress–response cascades, antioxidant defense activation, and apoptosis‐related mechanisms. Additionally, the emphasis on “bioavailability” reflects increasing efforts to understand how metal speciation and cellular uptake influence cellular toxicity, while the frequent use of “in vitro” underscores the reliance on mechanistic cellular models to investigate molecular endocrine, neurotoxic, and cytotoxic pathways.

Together, these keyword trends demonstrate that the scientometric landscape is progressively shifting toward detailed mechanistic studies, bridging publication patterns with cellular toxicity pathways, oxidative imbalance, genotoxic effects, and organ‐specific toxicological outcomes.

A research gap refers to an unexplored or insufficiently studied area within a field highlighting unanswered questions or unresolved issues. Identifying these gaps in a review article is crucial, as it not only emphasizes the limitations of current knowledge but also justifies the need for further studies and guides innovation. In the case of metal toxicity, several critical research gaps remain. Most studies have concentrated on well‐known toxic metals such as cadmium, lead, and mercury, while the toxicity of emerging and less‐studied metals, including rare earth elements (indium, gallium, thallium), cobalt, and titanium, remains poorly understood. Specifically, their potential to cause metal‐induced oxidative stress pathways has not been adequately explored. These metals are increasingly used in electronics such as semiconductors, photovoltaics, medical devices, and renewable energy technologies [60], raising concerns about long‐term ecological and health risks. These understudied metals may pose significant risks to aquatic ecosystems through bioaccumulation and food‐web transfer [61].

A recent investigation by Sarkar et al. [62] demonstrated that the coexposure of lead and chromium intensifies oxidative stress and hepatotoxicity via the Nrf2‐Keap1‐ARE pathway, highlighting the environmental and health risks associated with heavy metal contamination and also underscores the importance of redox regulation in metal hepatotoxicity. Future research should prioritize systematic toxicological assessments of these elements, focusing particularly in relation to chronic low‐dose heavy metal exposure pathways. Moreover, the integration of multiomics approaches such as genomics, transcriptomics, proteomics, and metabolomics with environmental monitoring will assist in generating a systems‐level understanding of metal pollutant toxicity. Nanobioremediation and biochar‐based amendments offer eco‐friendly and sustainable approaches with high adsorption and immobilization capacity, while microbial consortia embedded in polymeric carriers provide efficiency in complex environments. Genetically enhanced phytoremediation offers efficient site‐specific detoxification of metals. In addition, coupling remediation technologies with IoT‐enabled monitoring and machine learning models facilitates predictive and adaptive solutions. Current research clearly exhibits an ecosystem bias. Research has largely focused on urban‐industrial environments and freshwater systems, while estuaries, rural groundwater aquifers, Arctic regions, and deep‐sea sediments remain neglected. These ecosystems are critical reservoirs for toxic metals and play key roles in global biogeochemical cycles. Expanding research into these neglected environments will provide a more comprehensive understanding of contamination patterns and risks [63]. Added to this, methodological limitations continue to hinder progress. Cross‐study comparisons are hindered by the reliance on laboratory‐based, nonstandardized detection methods, highlighting the need for universally accepted protocols and the development of real‐time, portable, and cost‐effective biosensors for consistent field monitoring. Metallic pollutants exhibit dose‐dependent toxicological outcomes, with both acute high‐dose exposures and chronic low‐dose bioaccumulation contributing to adverse health effects [3]. Understanding dose–response relationships is essential for setting regulatory thresholds, risk assessment models, and public health guidelines. However, toxicity studies remain disproportionately focused on acute or high‐dose exposures, while chronic, low‐dose exposures that mirror real‐world conditions are underexplored [64]. Future research should prioritize long‐term exposure models and examine cumulative, synergistic, and antagonistic effects of multiple contaminants. There are very few mechanistic insights into toxicity. However, the mechanisms underlying metal genotoxicity and epigenetic dysregulation remain poorly understood, despite widespread recognition of metal‐induced oxidative stress, enzyme inhibition, and mitochondrial dysfunction. Advanced molecular tools such as genomics, proteomics, and metabolomics are needed to unravel these mechanisms and identify reliable biomarkers of exposure [65]. Finally, despite the rapid growth of academic research, translation into policy and large‐scale remediation remains limited. Stronger interdisciplinary collaborations are required to bridge laboratory findings with practical applications.

Moreover, the temporal and thematic evolution of research on metal toxicity highlights a transition from basic toxicity assessments toward advanced remediation strategies, risk‐based monitoring, and policy‐relevant insights. Addressing the identified research gaps understudied metals, neglected ecosystems, methodological standardization, chronic and synergistic exposures, mechanistic understanding, and translational research will not only advance scientific knowledge but also align directly with global sustainability agendas. For example, improved remediation technologies support SDG 6 (Clean Water and Sanitation), ecosystem‐focused toxicity studies contribute to SDG 14 (Life Below Water), and SDG 15 (Life on Land), and mechanistic insights strengthen SDG 3 (Good Health and Well‐Being), and circular economy practices promote G 12 (Responsible Consumption and Production). To better forecast real‐world exposure scenarios, future research should incorporate toxicokinetic modeling, nanometal interaction studies and cumulative risk assessment frameworks. Furthermore, closer alignment between scientometric insights and public health policy development is essential to integrate research findings into regulatory standards, occupational safety guidelines, and sustainable industry practices.

## 6. Conclusion

This scientometric study has compiled and analyzed the research output on the toxicity of metallic pollutants using data from the WoS database. The research in this field has steadily increased over the years, with an exponential development phase beginning in 2018 and peaking in 2023. Contributions have been made by 108 authors from 56 countries, with China (26.4%), France (17.3%), and India (8.6%) emerging as the top contributors, while the United Kingdom leads in citation impact with 2395 citations. The research has been published in 59 journals, with Environmental Sciences and Pollution Research and Environmental Pollution being the most commonly utilized and highly cited. A total of 77 keywords were found, with “toxicity” occurring 80 times, followed by terms linked to heavy metals, degradation, removal, and water pollution, indicating significant research issues and areas of increasing interest. The subject analysis indicates that Environmental Science and Ecology account for nearly half of the research focus, supplemented by emerging disciplines such as toxicology, materials science, and public health. The study further highlights significant collaboration among institutions, with the Chinese Academy of Sciences contributing the highest number of publications (10) and citations (272). The funding analysis showed the National Natural Science Foundation of China (NSFC) as the primary contributor, with additional contributions from the European Union and other worldwide agencies. This study highlights significant knowledge gaps in topics such as the toxicity of understudied metals, monitoring approaches, and chronic exposure models. It suggests that future research focuses on building cost‐effective biosensors, investigating molecular pathways using omics technologies, and translating discoveries into long‐term policy frameworks. Overall, this scientometric analysis helps to improve additional scientific knowledge and effectively guide the researchers, policymakers, and practitioners to manage and remediate metallic contaminants for better environmental and public health outcomes.

## Funding

No funding was received for this manuscript.

## Conflicts of Interest

The authors declare no conflicts of interest.

## Data Availability

All data utilized for the study are available in the manuscript itself.
